# GSK-3β activation accelerates early-stage consumption of Hippocampal Neurogenesis in senescent mice

**DOI:** 10.7150/thno.43829

**Published:** 2020-08-01

**Authors:** Fei Liu, Na Tian, Hua-Qiu Zhang, Shi-Hong Li, Qiu-Zhi Zhou, Ying Yang, Jie Zheng, Jian-Zhi Wang

**Affiliations:** 1 Department of Pathophysiology, Key Laboratory of Ministry of Education for Neurological Disorders, School of Basic Medicine, Tongji Medical College, Huazhong University of Science and Technology, Wuhan 430030, China.; 2 Department of Human Anatomy, School of Basic Medicine, Binzhou Medical University, Yantai 264003, China.; 3 Department of Histology and Embryology, Key Laboratory of Ministry of Education of China for Neurological Disorders, School of Basic Medicine, Tongji Medical College, Huazhong University of Science and Technology, Wuhan 430030, China.; 4 Key Laboratory of Ministry of Education for Neurological Disorders, Department of Neurosurgery, Tongji Hospital, Tongji Medical College, Huazhong University of Science and Technology, Wuhan 430030, China.; 5 Co-innovation Center of Neuroregeneration, Nantong University, Nantong 226000, China.

**Keywords:** Glycogen synthase kinase-3β, Adult hippocampal neurogenesis, Senescence

## Abstract

Adult hippocampal neurogenesis (AHN) deficits contribute to the progression of cognitive impairments during accelerated senescence, with the mechanistic causes poorly understood. Glycogen synthase kinase-3β (GSK-3β) is a critical regulator in prenatal neurodevelopment. The present study aims to study whether and how GSK-3β regulates AHN during the accelerated senescence.

**Methods:** AHN and AHN-dependent cognition and GSK-3β were evaluated in 3- and 6-month senescence-accelerated mice prone 8 (SAM-P8) and senescence resistant 1 (SAM-R1) mice, respectively. GSK-3β was selectively overexpressed in wild-type mice using adeno-associated virus, or knocked-out by crossbreeding with GSK-3β floxed mice in the neural stem cells (NSCs) of Nestin-Cre mice, or pharmacologically inhibited with SB216763 in SAM-P8 mice. AHN was evaluated by BrdU-, DCX-staining and retrovirus-labeling.

**Results:** AHN transiently increased at 3-month, but dramatically dropped at 6-month of age in SAM-P8 mice with a simultaneous activation of GSK-3β at 3-month. Selective overexpression of GSK-3β in hippocampal NSCs of wildtype mice induced long-term AHN deficits due to an accelerated depletion of NSC pool, although it transiently increased the proliferation and survival of the newborn neurons. Pharmacologically inhibiting GSK-3β by SB216763 efficiently preserved AHN and improved contextual memory in 6-month SAM-P8 mice, while conditional knock-out of GSK-3β in NSCs impaired AHN.

**Conclusion:** Early-stage activation of GSK-3β in NSCs impairs AHN by accelerating the depletion of NSC pool, and pharmacological inhibition of GSK-3β is efficient to preserve AHN during the accelerated aging. These results reveal novel mechanisms underlying the AHN impairments during accelerated senescence and provide new targets for pro-neurogenic therapies for related diseases.

## Introduction

Adult hippocampal neurogenesis (AHN) originates from self-renewing and multipotent neural stem cells (NSCs) residing in the sub-granular zone (SGZ) of hippocampal dentate gyrus (DG) 1-5. AHN involves multiple stages, including the proliferation of NSCs, survival and early-stage differentiation of intermediate progenitor cells (IPCs), as well as the maturation of newborn neurons, which is highly comparable to the process of prenatal neurodevelopment 6. Adult-born hippocampal neurons endow DG circuits high plasticity and play critical roles in hippocampus-dependent cognition and emotions 7, 8.

Recent studies suggested the fact that AHN persists but drops during aging 9-12. This age-dependent decline of AHN can be further accelerated to contribute to hippocampus-dependent cognitive and emotional dysfunctions in neurodegenerative diseases, such as Alzheimer's disease (AD), the most common form of dementia characterized by accelerated aging 13-17. However, molecular mechanisms underlying the accelerated AHN decline during abnormal aging remain poorly understood.

Glycogen synthase kinase-3β (GSK-3β) is a serine/threonine kinase, highly expressed in the central nervous system 18, which plays crucial roles in shaping the migration, polarization and axon growth of newborn hippocampal neurons during fetal neurodevelopment 19. An upregulation of GSK-3β function has been widely observed in neurodegenerative diseases, especially in AD 20, 21. However, whether and how the GSK-3β activation contributes to the progressive decline of AHN during accelerated aging remains unknown.

In the present study, we found that GSK-3β activation in hippocampal neural stem cells induced a transient increase of AHN by over-consumption of the NSCs pool in mice with accelerated senescence, which lead to an eventual AHN deficit thus impaired spatial memory, while only partial inhibiting GSK-3β rescues AHN.

## Results

### SAM-P8 mice show an early-stage increase but long-term deficit of AHN

**Figure 1:**
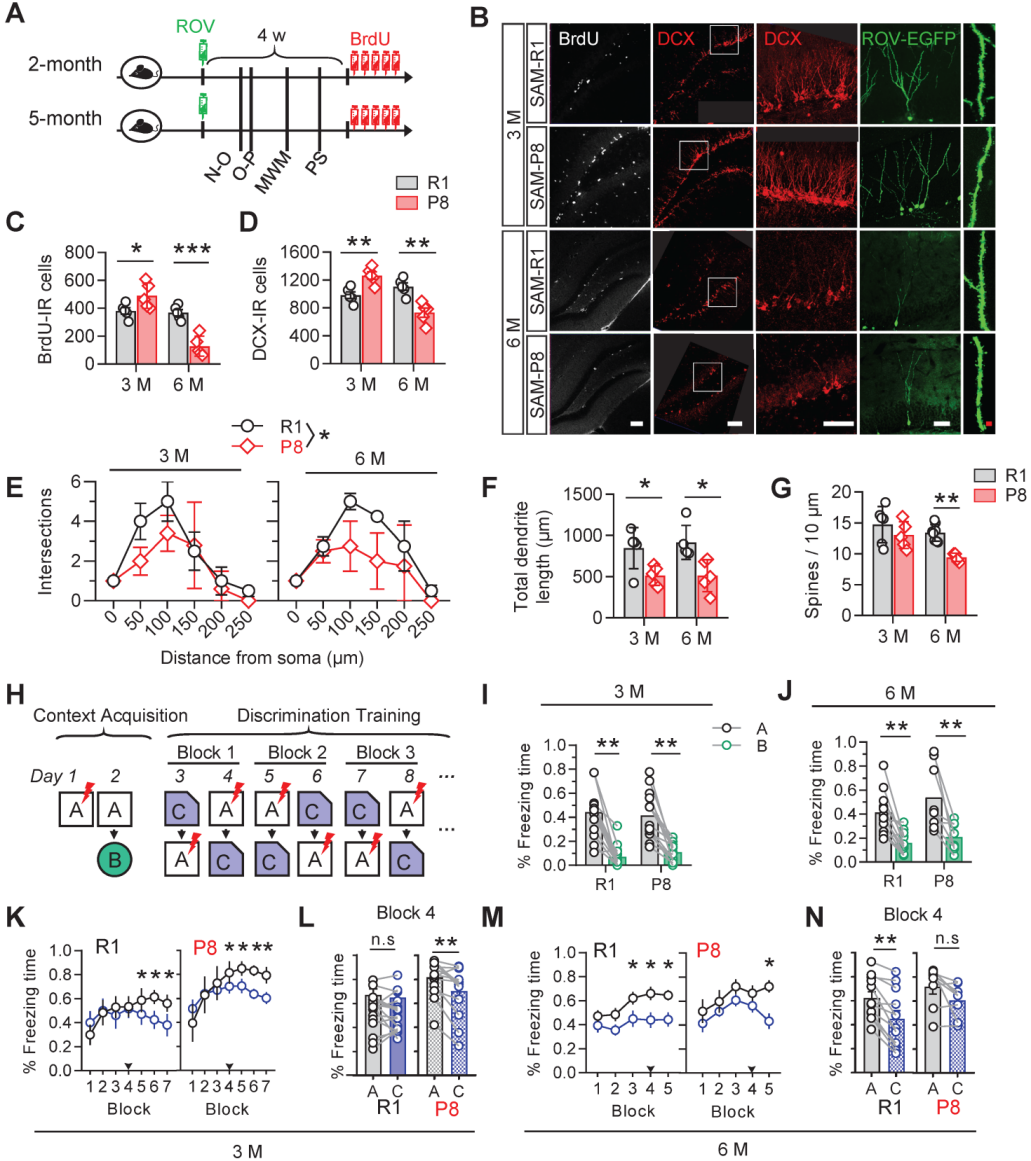
** SAM-P8 mice show transient increase but long-term deficits of AHN. (A)** Experimental scheme. Two or five months old SAM-P8 or R1 mice were stereotactically injected with ROV-GFP. Novel-object (N-O) recognition, object-place (O-P) recognition, spatial learning and memory in the Morris-water maze (MWM) and contextual pattern separation (PS) were tested in turn in the following month, and some mice were randomly selected for continuous intraperitoneal injection of BrdU before execution. **(B)** Representative images showing AHN in different ages of SAM-P8 or R1 mice. Scale bars were 50 µm (white) or 10 µm (red). **(C)** The number of BrdU- immunoreactive (IR) cells increased at 3-month of SAM-P8 mice but decreased at 6-month compared with age-matched R1 mice. Two-way ANOVA followed by Tukey's multiple comparisons tests, (genotype effect: F (1, 16) = 37.25, *P* < 0.0001; age effect F (1,16) = 4.574, *P* = 0.0482; interaction: F (1, 16) = 32.33, *P* < 0.0001). * *P* < 0.05, *** *P* < 0.0001. n = 5 mice/group.** (D)** The number of DCX-immunoreactive (IR) cells increased at 3-month of SAM-P8 mice but decreased at 6-month compared with age-matched R1 mice. Two-way ANOVA followed by Tukey's multiple comparisons tests (genotype effect: F (1, 16) = 13.48, *P* = 0.0021; age effect: F (1,16) = 0.6453, *P* = 0.4336; interaction: F (1, 16) = 34.25, *P* < 0.0001). ** *P* < 0.01. n = 5 mice/group. **(E)** SAM-P8 mice showed decreased dendrite complexity of ROV-GFP-labeled newborn neurons both at both 3- and 6-month compared with age-matched SAM-R1 mice. Repeated measures ANOVA followed by Tukey's multiple comparisons tests (3 M, genotype effect: F (1, 42) = 4.083, *P* < 0.05; distance effect: F (5,42) = 13.07, *P* < 0.0001; interaction F (5, 42) = 1.144, *P* = 0.3523. 6 M, genotype effect: F (1, 36) = 9.422, *P* < 0.0041; distance effect: F (5, 36) = 20.52, *P* < 0.0001; interaction: F (5, 36) = 1.426, *P* = 0.2384). **(F)** SAM-P8 mice showed decreased total length of ROV-GFP-labeled newborn neurons both at 3- and 6-month compared with age-matched SAM-R1 mice. Two-way ANOVA followed by Tukey's multiple comparisons tests (age effect: F (1, 16) = 0.1508, *P* = 0.7029; genotype effect: F (1,16) =17.42, *P* = 0.0007; interaction: F (1, 16) = 0.1473, *P* = 0.7062), * *P* < 0.05. **(G)** SAM-P8 mice showed decreased dendritic spine density of ROV-GFP-labeled newborn neurons at 6-month age. Two-way ANOVA followed by Tukey's multiple comparisons tests (age effect: F (1, 19) = 8.281, *P* = 0.0096; genotype effect: F (1,19) = 10.90, *P* = 0.0038; interaction: F (1, 19) = 1.737, *P* = 0.2032). ** *P* < 0.01. n = 5 mice/group. **(H)** Experimental scheme of behavioral training and testing procedures in the contextual acquisition and pattern separation.** (I-J)** Both SAM-P8 and SAM-R1 mice performed well in distinguishing context A from B, at both 3- and 6-month age. Paired t tests, ** *P* < 0.01. n = 8~16 mice/group. **(K-L)** SAM-P8 mice learned more quickly than age-matched SAM-R1 mice in discriminating context C from A at 3-month age (K). Especially, at block 4, SAM-P8 mice spent less time (L left), but R1 mice spent statistically equal time freezing in context C than A (L right), Repeated measures ANOVA followed by Tukey's multiple comparisons tests (R1, context effect: F (6, 210) = 4.444, *P* = 0.0003; block effect: F (1, 210) = 8.430, *P* = 0.0041; interaction: F (6, 210) = 3.260, *P* = 0.0044. P8, context effect: F (6, 168) = 6.875, *P* < 0.0001; block effect: F (1, 168) = 6.731, *P* = 0.0103; interaction: F (6, 168) = 1.907, *P* = 0.0824). (K) and Paired t tests (L), * *P* < 0.05, ** *P* < 0.01. n = 16 (R1) or n = 13 (SAM-P8) mice. **(M-N)** SAM-P8 mice at 6-month age learned more slowly than age-matched SAM-R1 mice in discriminating context C from A (M). Especially, at block 4, SAM-P8 mice spent statistically equivalent time (N left), but R1 mice spent less time freezing in context C than A (N right). Repeated measures ANOVA followed by Tukey's multiple comparisons tests (R1, context effect: F (4, 110) = 2.720, *P* = 0.0332; block effect: F (1, 110) = 21.20, *P* < 0.0001; interaction: F (4, 110) = 0.5407, *P* = 0.7061. P8: context effect: F (4, 70) = 2.377, *P* = 0.0601; block effect: F (1, 70) = 10.51, *P* = 0.0018; interaction: F (4, 70) = 0.7815, *P* = 0.5410). (M) and Paired t tests (N), * *P* < 0.05, ** *P* < 0.01. n = 12 (R1) or n = 8 (SAM-P8) mice. Data were represented as mean ± SEM.

We firstly examined the changes of AHN along with aging in age-matched senescence accelerated mice prone 8 (SAM-P8) and senescence resistant 1 (SAM-R1). The eGFP gene-carried retrovirus (ROV-eGFP) was injected stereotaxically into the hippocampal dentate gyrus (DG) for evaluating the dendrite maturation of the newborn neurons. Mice behaviors were tested in turn in the following month after retrovirus injection. 5-Bromo-2-Deoxyuridine (BrdU, 50 mg/kg) was injected intraperitoneally for 5 consecutive days before sacrificing the mice for the measurement of cell proliferation. Doublecortin (DCX) was used as a marker of immature neurons (Figure 1A).

Increased numbers of BrdU- and DCX-immunoreactive cells were detected in 3-month SAM-P8 mice compared with the age-match SAM-R1 mice, with decreases of both dendrite length and complexity but nonsignificant change of spine density of EGFP-labeled newborn neurons in SAM-P8 mice (Figure 1B-G). By contrast, in 6-month SAM-P8 mice, the cell numbers of both BrdU- and DCX-immunoreactive, as well as the dendrite complexity, length and spine density of newborn neurons were all decreased compared with age-matched SAM-R1 mice (Figure 1B-G).

In consistent with the above age-dependent alterations of neurogenesis, the expression level of synaptic proteins, including synapsin1, synaptotagmin or synaptophysin, in DG was also upregulated in 3-month but downregulated in 6-month SAM-P8 mice (Figure S1).

AHN plays important roles in contextual pattern separation 22. We observed that SAM-P8 mice performed well in discriminating very distinct contexts at both 3- and 6-month of age (Figure 1H-J), whereas in the paradigm to discriminate the contexts sharing much more common features, SAM-P8 mice learned more quickly at 3-month but more slowly at 6-month of age than age-match SAM-R1 mice (Figure 1K-N). Moreover, SAM-P8 mice at 6-month, but not 3-month of age also showed impairments in spatial learning and memory (Figure S2).

Taken together, these data indicated a transient increase of AHN number but subsequently multi-stage deficits of AHN in SAM-P8 mice.

### Increased activation of GSK-3β in NSCs of SAM-P8 mice

GSK-3β plays crucial roles both in shaping fetal neurogenesis 18, 22 and neurodegeneration in the elderly 23. To test the potential contribution of GSK-3β to the age-dependent alterations of AHN during accelerated senescence, we examined the activity-dependent expression levels of GSK-3β isoforms in the hippocampal DG subset of SAM-P8 and R1 mice.

**Figure 2:**
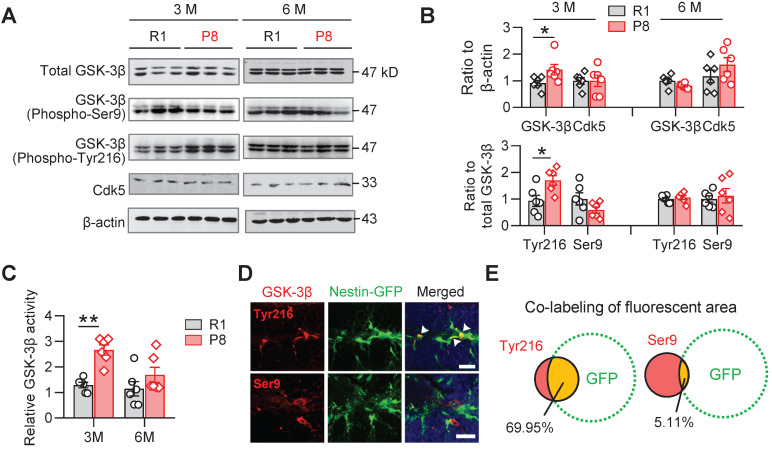
** SAM-P8 mice show transiently increased activation of GSK-3β in the DG at 3-month age. (A-B)** Upregulated expression of GSK-3β (phospho-Tyr216) and total GSK-3β in the DG of 3-month, but not 6-month SAM-P8 mice compared with age-matched R1 mice. Limited change of GSK-3β (phospho-Ser9) and Ckd5 were detected. Two-way ANOVA followed by Tukey's multiple comparisons tests (B above, genotype effect: F (1, 40) =2.286, *P* = 0.1384; age effect: F (3, 40) =2.968, *P* = 0.0432; interaction: F (3, 40) = 1.880, *P* = 0.1484. B below, genotype effect: F (3, 40) =3.066, *P* = 0.0388; age effect: F (1, 40) =1.205, *P* = 0.2789; interaction: F (3, 40) = 3.934, *P* = 0.0150). * *P* < 0.05, n = 6 mice/group. **(C)** Enhanced GSK-3β activity in the DG of 3-month but not 6-month SAM-P8 mice. Two-way ANOVA followed by Tukey's multiple comparisons tests (age effect: F (1, 20) = 5.649, *P* = 0.0276; genotype effect: F (1, 20) = 16.78, *P* < 0.0006; interaction: F (1, 20) = 3.127, *P* = 0.0923), ** *P* < 0.01, n = 6 mice/group. **(D-E)** High expression of GSK-3β (phospho-Tyr216) but not GSK-3β (phospho-Ser9) was observed in nestin-positive neural stem cells in Nes-GFP mice. Arrows indicate the co-labeling areas. Scale bars, 50 µm. n = 3 mice/group.

In 3-month, but not 6-month SAM-P8 mice, we observed increases of both total and active GSK-3β (i.e. phospho-Tyr216), with statistically unchanged levels of either inactive GSK-3β (phospho-Ser9) or Cdk5, another kinase involved in the regulation of AHN 21 (Figure 2A-B). Meanwhile, GSK-3β activity assay also revealed increased activation of GSK-3β in 3-month, but not 6-month SAM-P8 mice (Figure 2C). Further studies revealed a prominent localization of the active form of GSK-3β in nestin-positive NSCs (Figure 2D-E), suggesting a link of GSK-3β overactivation to the dysregulated AHN during accelerated senescence.

### NSC-overexpression of GSK-3β induces transient increase but long-term deficit of AHN

**Figure 3:**
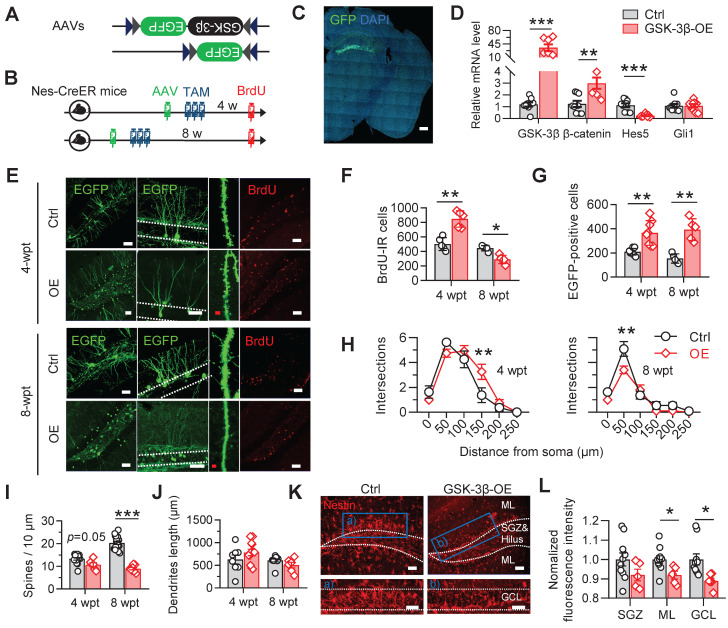
** Selective overexpressing GSK-3β in NSCs induces temporal increase but long-term deficits of AHN. (A)** AAV-EF1α-DIO-GSK-3β-2A-EGFP or AAV-EF1α-DIO-EGFP was stereotaxically infused into the DG for selective overexpressing GSK-3β in NSCs. **(B)** Experimental scheme. Tamoxifen (TAM) was injected for 3 consecutive days following AAVs injection, and BrdU was injected before sacrificing the mice. **(C)** Representative image showing the GFP expression by AAVs in the DG. Scale bar, 300 μm. **(D)** GSK-3β-2A-EGFP overexpression upregulated GSK-3β and β-catenin mRNA levels, with downregulated Hes5 but unchanged Gli1 mRNAs in EGFP-positive cells. Unpaired t tests. ** *P* < 0.01, *** *P* < 0.001. n = 3 mice/group. **(E)** Representative images showing AHN at 4- or 8-weeks post AAVs injection and TAM (wpt) administration. Scale bars were 50 μm (white) or 10 μm (red). **(F)** Overexpressing GSK-3β increased the number of BrdU-labeled cells at 4-wpt, but decreased BrdU-labeled cells at 8-wpt. Two-way ANOVA followed by Tukey's multiple comparisons tests (virus effect: F (1, 16) = 74.86, *P* < 0.0001; time effect: F (1, 16) = 7.268, *P* = 0.0159; interaction: F (1, 16) = 49.70, *P* < 0.0001). * *P* < 0.05, ** *P* < 0.01. n = 5 mice/group. **(G)** Overexpressing GSK-3β increased the number of EGFP-positive newborn cells both at 4- and 8-wpt. Two-way ANOVA followed by Tukey's multiple comparisons tests (time effect: F (1, 22) = 0.2388, *P* = 0.6299; virus effect: F (1, 22) = 45.15, *P* < 0.0001; interaction: F (1, 22) = 2.032, *P* = 0.1681). ** *P* < 0.01. n = 5 mice/group. **(H)** Overexpressing GSK-3β increased the dendrite complexity of EGFP-labeled neurons at 4-wpt, especially at the distal part, but reduced the complexity of proximal dendrites at 8-wpt. Two-way ANOVA followed by Tukey's multiple comparisons tests (4-wpt, virus effect: F (1, 108) = 1.053, *P* = 0.3071; distance effect: F (5, 108) = 57.50, *P* < 0.0001; interaction: F (5, 108) = 3.099, *P* = 0.0118. 8-wpt, virus effect: F (1, 114) = 60.80, *P* < 0.0001; distance effect: F (5, 114) = 8.511, *P* = 0.0042; interaction: F (5, 114) = 3.085, *P* = 0.0119). ** *P* < 0.01. n = 5 mice/group. **(I)** Overexpressing GSK-3β reduced the dendritic spine density of EGFP-labeled newborn neurons at 8-wpt. Two-way ANOVA followed by Tukey's multiple comparisons tests (virus effect: F (1, 44) = 12.52, *P* = 0.001; time effect: F (1, 44) = 87.60, *P* < 0.0001; interaction: F (1, 44) = 35.09, *P* < 0.0001). *** *P* < 0.001. n= 8~19 neurons/group. **(J)** Overexpressing GSK-3β did not change the total dendrite length of EGFP-labeled newborn neurons both at 4- and 8-wpt. Two-way ANOVA followed by Tukey's multiple comparisons tests (virus effect: F (1, 32) = 0.1322, *P* = 0.7185; time effect: F (1, 32) = 4.145, *P* = 0.0501; interaction: F (1, 32) = 3.244, *P* = 0.0811). n= 8-12 neurons/group. **(K-L)** GSK-3β overexpression in NSCs dominantly decreased the pool of nestin-positive radial NSCs in the DG granular cell layer (GCL) and molecular layer (ML). Scale bars were 50 μm (L). SGZ, subgranular cell zone. Unpaired t tests, * *P* < 0.05. n= 4 mice/group.

To test how the GSK-3β overactivation in NSCs interferes AHN, we selectively overexpressed human GSK-3β in NSCs by stereotaxically infusing a double-floxed inverted open reading frame (DIO)-contained adeno-associated virus (i.e. AAV-EF1α-DIO-GSK-3β-2A-EGFP) into the DG of nestin-Cre/ERT2 mice, a transgenic line in which the tamoxifen-inducible Cre expression is restricted within NSCs (Figure 3A-C). AAV-EF1α-DIO-EGFP was injected as a control. After virus injection, tamoxifen (0.2 mg/g, dissolved in corn oil) was injected intraperitoneally for 3 consecutive days to induce Cre-mediated gene recombination (Figure 3B). Efficient GSK-3β overexpression in NSCs was confirmed by the dominantly upregulated mRNA of GSK-3β, with upregulated β-catenin and downregulated Hes5, two downstream genes of GSK-3β signaling cascades (Figure 3D).

GSK-3β overexpression significantly increased the number of BrdU-labeled newborn cells increased 4 weeks post tamoxifen (wpt), but decreased the BrdU-labeled cell number 8-wpt (Figure 3F), suggesting a temporal increase, but subsequent decrease of AHN. Though GSK-3β overexpression tended to facilitate the survival of NSC-derived newborn cells, as indicated by increased number of EGFP-labeled newborn cells at both 4- and 8-wpt (Figure 3G), and increase distal dendrites complexity EGFP-labeled newborn neurons at 4-wpt, it hindered the dendrite maturation of newborn neurons 8-wpt as shown by the decrease of both dendrite complexity (Figure 3H) and spine density (Figure 3I) of mice with GSK-3β overexpression, though no statistical change in the total dendrite length was observed both 4- and 8-wpt (Figure 3J).

Importantly, we also found that overexpressing GSK-3β in NSCs at 4-wpt significantly decreased the proportion of nestin-positive radial NSCs in the DG subgranular cell zone (SGZ) and granular cell layer (GCL) (Figure 3K-L), suggesting an increased consumption of quiescent NSCs, in consistent with recent findings that the consumption of NSC pool was accelerated in young SAM-P8 mice 24.

These data together suggested that specific GSK-3β-overexpression in NSCs temporarily promotes newborn neurons production, but induces eventually AHN deficits by accelerating the consumption of NSC pool.

### Inhibiting GSK-3β by SB216763 rescues AHN and improves cognitive functions in 6-month SAM-P8 mice

**Figure 4:**
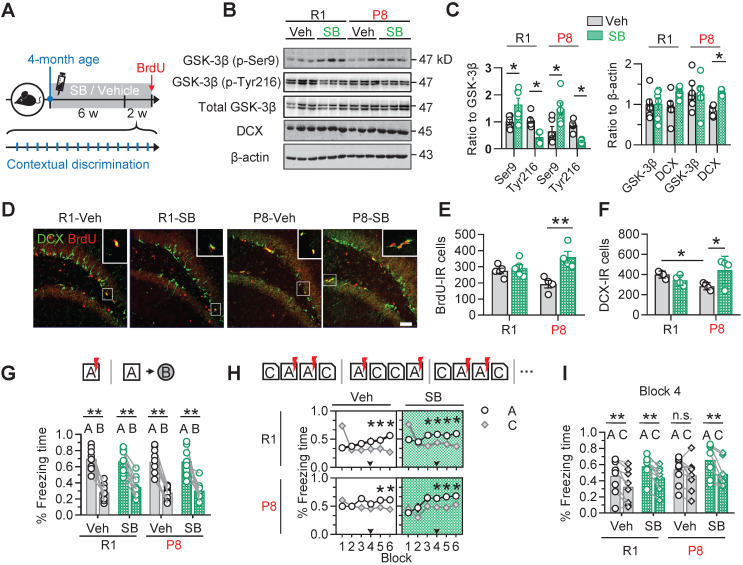
** Pharmacologically inhibiting GSK-3β by SB216763 preserves AHN and improves contextual pattern separation in 6-month SAM-P8 mice. (A)** Experimental scheme of SB216763 (SB) administration and contextual training. **(B-C)** SB upregulated the expression of inactive GSK-3β (phospho-Ser9) and downregulated the expression of active GSK-3β (phospho-tyr216) in both SAM-P8 and SAM-R1 mice of 6-month age, while only increased DCX expression in SAM-P8 mice. Unpaired t tests, * *P* < 0.05, n = 6 mice/group. **(D-F)** SB significantly increased both the BrdU- and DCX-positive cells numbers in 6-month SAM-P8, but not in SAM-R1 mice. Two-way ANOVA followed by Tukey's multiple comparisons tests (panel E, drug effect: F (1, 14) = 0.08714, *P* = 0.7722; genotype effect: F (1, 14) = 14.23, *P* = 0.0021; interaction: F (1, 14) = 9.709, *P* = 0.0076). Panel F, drug effect: F (1, 12) = 0.04340, *P* = 0.8385; genotype effect: F (1, 12) = 1.834, *P* = 0.2007; interaction: F (1, 12) = 7.910, *P* = 0.0157). * *P* < 0.05, ** *P* < 0.01, n = 4 mice/group. **(G)** Both SAM-P8 and R1 mice of 6-month age performed well in distinguishing very distinct contexts (context A and B), which was not significantly affected by SB. Paired t tests, ** *P* < 0.01. n = 9 mice/group. **(H)** SB administration promoted the pattern separation in both SAM-P8 and R1 mice during discriminating the pair of similar contexts (context A and C). Repeated measures ANOVA followed by Tukey's multiple comparisons tests (left above, context effect: F (5, 84) = 2.779, *P* = 0.0226; block effect: F (1, 84) = 2.838, *P* = 0.0957; interaction: F (5, 84) = 7.567, *P* < 0.0001. right above, context effect: F (5, 84) = 3.880, *P* = 0.0033; block effect: F (1, 84) = 8.550, *P* = 0.0044; interaction: F (5, 84) = 7.350, *P* < 0.0001. left below, context effect: F (5, 96) = 0.3661, *P* = 0.8706; block effect: F (1, 96) = 4.315, *P* = 0.0404; interaction: F (5, 96) = 1.608, *P* = 0.1652. right below, context effect: F (5, 94) = 4.148, *P* = 0.0019; block effect: F (1, 94) = 11.51, *P* = 0.001; interaction: F (5, 94) = 1.118, *P* = 0.3562), * *P* < 0.05. n = 9 mice/group. **(I)** At block 4, SAM-P8 mice administrated with SB but not vehicle spent less time freezing in context C than A. Paired t tests, ** *P* < 0.01. n = 9 mice/group.

To confirm the contribution of active GSK-3β upregulation to the AHN deficits in SAM-P8 mice, we injected SB216763 (SB) (intraperitoneal, 2 mg/kg), a specific and competitive inhibitor of GSK-3β 25, 26, into 4-month old SAM-P8 mice once every other day for 6 consecutive weeks (Figure 4A). The inhibition of GSK-3β by SB216763 was confirmed by the upregulated level of inactive GSK-3β (phospho-Ser9) and downregulated level of inactive GSK-3β (phospho-Tyr216) in the DG subset compared with vehicle (Veh) (Figure 4B-C). Prior administration of SB216763 remarkably reversed the AHN deficits in 6-month SAM-P8 but not SAM-R1 mice, shown by the increased number of BrdU- and DCX-labeled cells (Figure 4D-F). Additionally, application of SB216763 also upregulated the expression of synapsin1 and PSD95 in both SAM-P8 and R1 mice at 6-month of age (Figure S3). In consistent with the improved AHN and expression of synapse-associated proteins, inhibiting GSK-3β by SB216763 also improved mice performance in the contextual pattern separation of SAM-P8, but not SAM-R1 mice at the 6-month of age (Figure 4G-I).

### Genetic knock-out of GSK-3β in NSCs impairs AHN in normal mice

**Figure 5:**
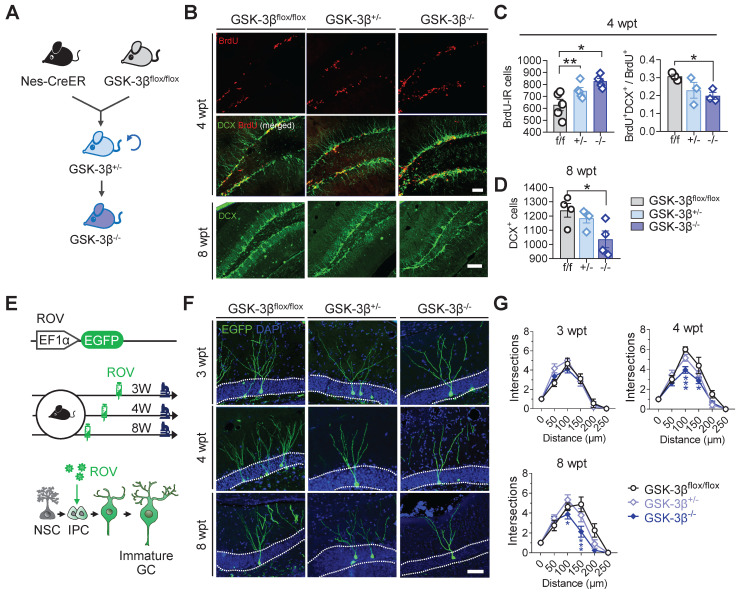
** Conditional GSK-3β knock-out in NSCs induces a time-dependent AHN deficit. (A)** GSK-3β was conditionally knocked-down in NSCs by cross-breeding Nes-Cre/ERT2 with GSK-3β^flox/flox^ mice, and then knocked-out by cross-breeding GSK-3β^+/-^ mice. **(B)** Representative immunofluorescent images showing BrdU- and DCX- labeled AHN at 4- and 8-wpt. Scale bars were 50 μm. **(C)** Both GSK-3β knock-down and knock-out increased the number of BrdU-labeled newborn cells at 4-wpt (left), while GSK-3β knock-out significantly decreased the proportion of BrdU/DCX co-labeled cells (i.e. immature newborn neurons). One-way ANOVA followed by Tukey's multiple comparisons tests (Left, F (2,12) = 8.301, *P* = 0.0055. Right, F (2,6) = 3.750, *P* = 0.0878). * *P* < 0.05, ** *P* < 0.01. n= 3~5 mice/group. **(D)** GSK-3β knock-out but not knock-down reduced DCX-positive immature neurons at 8-wpt. One-way ANOVA followed by Tukey's multiple comparisons tests (F (2, 9) = 4.917, *P* = 0.0361). * *P* < 0.05, n= 3~5 mice/group. **(E)** Experimental scheme illustrating the labeling of newborn neurons with ROV-EGFP. **(F)** Representative images showing the dendrite maturation of ROV-EGFP-labeled newborn neurons. **(G)** GSK-3β^-/-^ mice exhibited decreased dendrite complexity of newborn neurons both at 4- and 8-wpt but not at 3-wpt, compared with the age-matched GSK-3β^flox/flox^ or GSK-3β^+/-^ littermates. Repeated measures ANOVA followed by Tukey's multiple comparisons tests (3-wpt, genotype effect: F (5, 156) = 76.29, *P* < 0.0001; distance effect: F (2, 156) = 0.7106, *P* = 0.4929; interaction: F (10, 156) = 1.036, *P* = 0.4155. 4-wpt, genotype effect: F (5, 156) = 112.9, *P* < 0.0001; distance effect: F (2, 156) = 11.44, *P* < 0.0001; interaction: F (10, 156) = 2.609, *P* = 0.0059. 8-wpt, genotype effect: F (5, 132) = 54.16, *P* < 0.0001; distance effect: F (2, 132) = 6.314, *P* = 0.0024; interaction: F (10, 132) = 2.700, *P* = 0.0048). * *P* < 0.05. n = 3 mice/group. Data were represented as mean ± SEM.

To further verify the physiological role of NSC-GSK-3β in AHN, we produced NSC-conditional GSK-3β knock-out mice by crossbreeding nestin-Cre/ERT2 with GSK-3β-floxed mice and treated the mice with tamoxifen (Figure 5A). We observed unexpectedly that NSC-specific GSK-3β knock-out significantly increased BrdU-labeled newly generated cells in the hippocampal DG subset at 4-wpt (Figure 5B-C). By further analysis, we observed a decreased portion of BrdU and DCX co-labeled cells at 4-wpt (Figure 5B-C), suggesting that those BrdU-labeled cells were unlikely to differentiate into neurons. This point was further confirmed by the decreased number of DCX-positive cells in GSK-3β^-/-^ mice at 8-wpt, compared with the littermates of GSK-3β^flox/flox^ or GSK-3β^+/-^ (Figure 5D). In addition, GSK-3β knock-out also impaired the late-stage dendrite differentiation of newborn neurons, as evidenced by the decreased dendrite complexity of ROV-GFP-labeled newborn neurons both at 4- and 8-wpt, but not at 3-wpt (Figure 5E-G). These data together suggest that a baseline level of GSK-3β is essential for shaping the neuronal differentiation and dendrite maturation of the newborn neurons, though NSC GSK-3β-overexpression impairs AHN.

## Discussion

AHN was firstly reported in 1965 to decline along with increased age 27. Although newborn hippocampal neurons in normally aged brains were scarce and exhibited slow development, they displayed a remarkable potential for plasticity 10. Recent findings suggested that AHN in human brains can generally persist into elderly 5, 11, but the age-dependent decline of AHN could be further accelerated under neurodegenerative diseases, such as AD 16, 17. The maintenance of AHN depends on a tight balance between the self-renewal and consumption of NSC pool. However, an increased early-stage consumption of NSCs was reported both in the accelerated senescence and pre-symptomatic AD 24, 28. As a result, in transgenic AD animals, which showed accelerated aging and neurodegenerative symptoms 29-31, newly-born hippocampal neurons tended to transiently increase in number, but exhibit long-term deficits both in number and dendrite maturation. Consistently, we found in the present study that AHN increased in number at 3-month, but was impaired at 6-month SAM-P8 mice.

GSK-3β is a key regulator in controlling the neural progenitor homeostasis during the fetal development of mammalian brains 32. GSK-3β deletion was reported to have caused the hyperproliferation of neural progenitors along the neuraxis, but suppressed the generation of both intermediate neural progenitors and postmitotic neurons, by dysregulating either the β-catenin, sonic hedgehog, notch or fibroblast growth factor signaling cascades 18. Constitutive activation of GSK-3β could also reduce the number of neural progenitors and inhibit axon formation during the prenatal neurogenesis 33-35. In adulthood, GSK-3β was reported to show abnormal overactivation and accumulation in neurodegenerative disorders like AD 36. Overexpression of GSK-3β in mature excitatory neurons (CaMKIIα-positive) in mouse DG altered the dendritic maturation of newborn neurons 37. Specific knockdown of Disc 1, an upstream inhibitor of GSK-3β, in neural progenitors also led to a premature neuronal differentiation at the expense of accelerated depletion of progenitors pool 38. However, direct evidence of whether and how intracellular accumulation of GSK-3β in NSCs determines AHN during accelerated aging remains elusive. In the present study, we reported an early-stage increase of GSK-3β activation in the hippocampal neurogenic niche of mice with accelerated senescence, and further evidenced by specific overexpression of GSK-3β in NSCs that GSK-3β gain-of-function induced long-term AHN deficits presumedly through accelerating the consumption of NSCs pool. It should be noted that although NSC-derived newborn cells transiently increased at earlier stages post GSK-3β-overexpression, these cells did not exhibit typical neuron-like morphologies, possibly as a result of deficits in the establishment of neuronal polarity 34.

The observed AHN deficits induced by GSK-3β overactivation could be at least partly attributed to the dysregulation of downstream wnt/β-catenin and notch signaling cascades, both of which had been reported to play important roles in the fate determination of intermediate neural precursor cells during adult neurogenesis 39, 40. Both Wnt ligands and overexpression of β-catenin was found to have induced the neuronal differentiation of primary human glioma stem cells presumedly by suppressing Notch signaling 41, which is essential for maintaining the proliferative and undifferentiated state of neural progenitor cells 42-45. Here, we found that GSK-3β overexpression upregulated the mRNA of β-catenin, but downregulated the mRNA of hes5 in NSCs.

Nevertheless, other mechanisms underlying the GSK-3β-induced AHN deficits should not be excluded. For instance, GSK-3β overactivation or accumulation might accelerate the depletion of NSC pool and impair AHN through inducing the hyperphosphorylation of tau, a microtubule-associated protein plays pivotal roles in shaping AHN 46-48, since tau hyperphosphorylation has been widely accepted as one of hallmarks of accelerated aging and neurodegenerative diseases 49. We have recently evidenced in a parallel study that accumulation of phosphorylated tau in GABAergic interneurons in the neurogenic niche induced AHN deficits, by disinhibiting local neural circuits and increasing NSC-derived astrogliosis 50. However, whether and how tau hyperphosphorylation by GSK-3β in NSCs determines AHN, especially during accelerated senescence, remains unknown.

Importantly, we found here that the AHN deficits in senescence mice were efficiently rescued by a pharmacological inhibitor of GSK-3β, SB216763, which also improved the contextual pattern separation of SAMP8 mice. In similar, AR-A014418, another specific inhibitor of GSK3β, was also reported to be efficient in preventing the reduction of neurogenic cortical progenitor cells and the decrease of neurogenesis induced by intraventricular hemorrhage 33. These results suggested a potential of GSK-3β inhibitors as pro-neurogenic agents for the treatment of neurodegenerative diseases.

Our results showed that although GSK-3β knock-out in neural progenitors promoted the proliferation of stem cells, the newly generated cells did not differentiate into new neurons. As a result, the number of newborn neurons decreased, and the dendritic development of immature neurons was also significantly impaired. These results are consistent with findings in embryos that GSK-3β depletion resulted in the hyperproliferation of neural progenitors along the entire neuraxis but suppressed the generation of intermediate neural progenitors and postmitotic neurons 18. However, a moderate reduction but not deletion of GSK-3β can effectively maintain the neural stem cell pool and induce long-term neurogenesis. These results indicate an important role of the balance between GSK-3β inhibition and activation in determining the neural stem cell proliferation and the neuronal differentiation of progenitor cells.

In conclusion, we found in the current study that GSK-3β activation in the NSCs of mice with accelerated senescence expedited the consumption of AHN pool, resulting in a transient increase but induced long-term AHN deficits and associated cognitive impairments. These results revealed novel mechanisms underlying the decline of neurogenesis during accelerated aging, and provided new targets for pro-neurogenic therapies for neurodegenerative diseases.

## Material and Methods

### Animals

The senescence accelerated mouse prone 8 (SAM-P8) and resistant 1 (SAM-R1) were purchased from the Department of Laboratory Animal Science, Peking University Health Science Center. C57BL/6-Tg (Nes-cre/ERT2) KEisc/J (termed as Nestin-Cre/ERT2) mice were obtained from the Jackson Laboratory. The GSK-3β floxed mouse was a kind gift from Dr. James R Woodgett 51, Nestin-GFP mice were obtained from Cyagen (Suzhou, China). All mice were housed under standard conditions under a 12-h light/dark cycle, with food and water ad libitum. Only male mice weighing 20 ~ 30 g were used in all experiments. All animal studies were approved by the Ethics Committee of Tongji Medical College, Huazhong University of Science and Technology.

### Viruses and stereotaxic injection

AAV-EF1α-DIO-GSK-3β-2A-EGFP and AAV-EF1α-DIO-EGFP was packaged by BrainVTA (Wuhan, China). pROV-EF1α (S)-EGFP (ROV-EGFP) were purchased from OBiO (Shanghai, China) (Cat# CN889). For virus injection, mice were anesthetized with ketamine (100 mg/kg) and xylazine (50 mg/kg), and then fixed in a stereotaxic apparatus (RWD, China). The scalp was sterilized with iodophors and incised along the skull midline, holes were drilled bilaterally and a total of 500 nL virus were delivered into each site of the dentate gyrus (posterior 1.9 mm, lateral ± 1.1 mm, and ventral 2.0 mm relative to bregma), using an automatic microinjection system (World Precision Instruments, USA), at a rate of 50 nL/min. The needle was kept in place for 10 min before being slowly pulled out. The skin was sutured and sterilized with iodophors. Mice were placed in thermos tank for analepsia.

### AHN evaluation

Fifty-μm brain sections were sliced using a vibrating microtome (VT1000S, Leica, Germany). Only AHN in the dorsal DG (approximately from AP -1.7 to -2.5) was evaluated in the present study. Immunofluorescent images were obtained by scanning a z series stack at a 3 μm interval throughout the entire 50 μm-thickness. NSC morphology was evaluated using Nes-cre/ERT2 mice. BrdU (B9285, Sigma-Aldrich) was dissolved in 0.01 M PBS (10 mg/ml) and injected (intraperitoneally, 50 mg/kg) for 5 consecutive days before execution for the evaluation of cell proliferation. The quantification of cell numbers was performed as described previously 52. Briefly, every fifth and a total of 3 sections were stained and then counted by an experimenter blinded from animal groupings. The cell counts were multiplied by 5 and added to indicate the total number of cells in the dorsal DG. Cell numbers in left and right DG were counted separately but averaged. The dendrite length and complexity of ROV-GFP-labeled newborn neurons were evaluated by Sholl-analysis using Simple Neurite Tracer plugin 53 and ImageJ (Fiji) software 54. Dendrite spines of ROV-GFP-labeled neurons were imaged under 100× oil immersion lens. Only secondary dendrites were analyzed in the present study.

### Western blotting

Western blotting was performed as described in our previous studies 55. In brief, mice were killed by overdose of ketamine and xylazine, and dentate gyrus was isolated as described as described by Hagihara et al 56. Proteins were extracted using RIPA buffer (P0013B, Beyotime). Protein concentration was determined by BCA method. An equal amount of total proteins for each sample was loaded, separated by 10% SDS-polyacrylamide gel electrophoresis, and transferred onto nitrocellulose membranes. The membranes were blocked with 5% defatted milk for 1 hour at room temperature, and subsequently incubated with primary and IRDye® 800CW-conjuncted secondary antibodies (1:2000, LI-COR Biosciences), in turn, overnight at 4 °C: GSK-3β (Phospho-Ser9) (1:1000, 9323S, Cell signaling), GSK-3β (Phospho-Tyr216) (1:1000,05-413, Millipore), GSK-3β (1:1000, 40988, Signalway Antibody), CDK5 (1:500, 33178, Signalway Antibody), DCX (1:1000, ab18723, Abcam), Synapsin1 (1:1000, ab64581, Abcam), synaptotagamin (1:1000, ab13259, Abcam), synaptophysin (1:1000, ab32127, Abcam), PSD95 (1:1000, 2507, Cell signaling), β-actin (1:1000, 21800, Signalway Antibody). The bands were visualized after washing with TBST using an Odyssey Infrared Imaging System (LI-COR biosciences, Lincoln, NE, USA). Bands were analyzed by Image J software (Fiji).

### GSK-3β activity assay

The activity of GSK-3β was assayed using a commercial kit (GMS50161.4, Germed), by following the manufacturer's instructions. In brief, mice were killed by overdose ketamine and xylazine. Proteins in hippocampal DG were extracted using GENMED lysis buffer (Reagent B), and the protein concentration was determined by BCA method. Buffer solution (Reagent C, 130 μL), enzymatic solution (Reagent D, 20 μL), reaction solution (Reagent E, 20 μL) and substrate solution (Reagent F, 20 μL) were incubated in 30 °C for 3 minutes in 96-well plate. Negative solution (Reagent G, 10 μL) or 50 μg protein sample were added into a 96-well plate, the absorbance was measured with microplate reader after 0 and 5 minutes, respectively. Relative GSK-3β activity were calculated according to the following formula: [(5 min absorbance - 0 min absorbance) × sample dilution factor × 0.1] / [0.005 × 6.22 (absorbance fraction) × 0.5 × reaction time].

### Immunofluorescence

Mice were anesthetized with ketamine (100 mg/kg) and xylazine (50 mg/kg), and transcardially perfused with normal saline, followed by 4% paraformaldehyde (PFA). Brains were removed and post-fixed for additional 48 h. Brains were sliced every 50 μm with a vibrating microtome (VT1000S, Leica, Germany). Sections were permeabilized in phosphate buffer containing 0.5% Triton x-100 for 30 min, blocked by 3% BSA for 30 min, and then incubated with primary antibodies overnight at 4 °C: GSK-3β (Phospho-Ser9) (1:100, 14630, Cell signaling), GSK-3β (Phospho-Tyr216) (1:200, 05-413, Millipore), BrdU (1:200, MCA2483, Bio-Rad), DCX (1:100, ab18723, Abcam), NeuN (1:400, MABN140, Millipore), Nestin (1:200, AP31810PU-N, Acris Antibodies). Brain sections were subsequently washed in PBS, and incubated with Alexa Fluor 546 or 488-conjuncted donkey secondary antibodies (1: 500, Invitrogen), respectively, for 1 h at room temperature. Images were obtained with a laser-scanning confocal microscope (710, Zeiss, Germany).

### Flow cytometry and real-time quantitative PCR (RT-qPCR)

AAV-EF1α-DIO-GSK-3β-EGFP or AAV-EF1α-DIO-EGFP was stereotaxically injected into the DG of Nestin-Cre/ERT2 mice. DG tissue was dissected 3 weeks after virus injection, digested with 0.125% trypsin and filtrated with a 30 μm filter to remove undissociated tissue and debris. GFP-positive cells were sorted using MoFlo (Beckman Coulter). Only GFP-positive cells were collected. RNA was extracted from collected samples, and cDNA was synthesized using random hexamers, oligodT15 and MultiScribe reverse transcriptase (Applied Biosystems). RT-qPCR was performed as previously described 57. Primers for qPCR were shown in Table S1.

### Contextual pattern separation

Mice were handled for 3 consecutive days before test, and then conditioned by a 2s 65 mA footshock in context A (i.e. the standard fear conditioning chamber), after 180s free exploring in the chamber at day 1. At the next day, mice were tested for fear generalization for 180 s, in turn, in the context A and a very distinct context (Context B, a 45×45×45 cm open field with Plexiglas floor and walls), both without footshock. Freezing behaviors of each mouse was recorded and analyzed online by an FCT-100 system (Techman, China).

In subsequent days, mice were trained through repeated trials to discriminate a pair of similar contexts with much more common features (i.e. context A and C). Context A was paired with footshock and characterized by an additional 65 dB white noise, and odorless 5% sodium hydroxide solution used to clean the chamber between animals. By contrast, context C was paired with green background lighting, a plastic ladder-shaped insert and 1% acetic acid odor, but without footshock. A pan filled with 0.25% benzaldehyde in alcohol was placed under the grid floor of the chamber in each trial to avoid the interference between different animals 58. The order of context A and C at each day during the training followed a sequence in pseudorandom. Freezing behavior was defined as behavioral immobility except for movement necessary for respiration Discriminative scores between context A and C were calculated as the freezing time (A - C) / (A + C), and averaged for every two days as the mean score in each block.

### Object-place and novel-object recognition

In object-place recognition test, mice were placed in a 50×50×50 cm plastic box with two identical objects (A and B) at two different locates, and allowed to freely explore for 5 min. 24 hours later, mice were placed in the box again and allowed to explore freely in the box for another 5 min, when object B (in pseudorandom) was removed to a new place while the object A remained at the same place as the day before. Mice behaviors were videotaped and analyzed using OFT-100 system (Techman, China). Bias scores toward object B were calculated as the exploring time (B-A)/(B+A).

In the novel object recognition test, a similar protocol was used, except that object B was replaced by a novel object (object C, a wooden cube) in the test phase, and mice preference for the novel object was measured.

### Morris water maze (MWM) test

MWM tests were performed as previously described 59. Briefly, mice were handled for 3 consecutive days before test, and then trained to find a hidden platform submerged under water, for 5 consecutive days, with 3 trials per day. Visual cues outside the pool remained constant. In each trial, mice were placed in one of the three quadrants without platform (in pseudorandom), facing pool wall and allowed to seek the hidden platform for 60 s, mice those failed to find the target were guided to the platform and placed for another 20 s. Travelling path of each mouse in each trail was recorded and analyzed online using an MWZ-100 system (Techman, China). At day 6, each mouse was tested for 60 s in the water maze with the hidden platform removed. Time stayed in the previous target quadrant and the number of target platform crossings was recorded.

### Statistical analysis

Data were presented as means ± SEM, unless otherwise specified. All data were analyzed and plotted using GraphPad Prism 8 software. Unpaired or paired two-tailed *t* tests, ANOVA (one-way, two-way or repeated measures) analysis and post hoc Tukey's multiple comparisons test were used for data analysis (as illustrated in figure legends), with *P* < 0.05 was considered as statistically significant.

## Supplementary Material

Supplementary figures and tables.
