# Supplementary material for: GSK-3β activation accelerates early-stage consumption of Hippocampal Neurogenesis in senescent mice
Source: Theranostics. 2020 Aug 1;10(21):9674–85. doi: 10.7150/thno.43829 (PMC7449917; doi:10.7150/thno.43829)
Supplement: Supplementary file 1 — Supplementary figures and tables. [file thnov10p9674s1.pdf]

Supplemental information

Figure S1

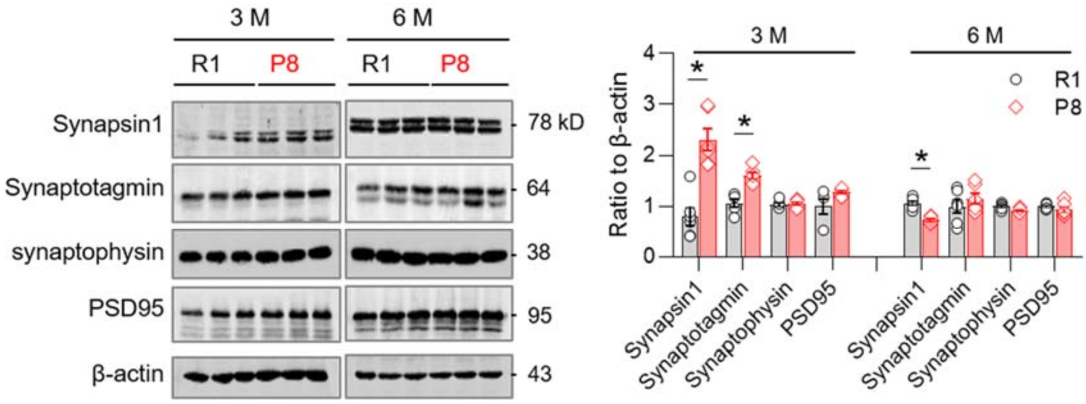

Figure S1. SAM-P8 mice exhibits an increased level of presynaptic proteins in DG at 3-month but a decreased level at 6-month. The levels of synapsin1 and synaptotagmin in the hippocampal DG were upregulated at 3-month SAM-P8 but downregulated at 6-month of SAM-P8 mice. PSD95 exhibited no statistical change in SAM-P8 mice at 3- and 6-month. Data were normalized to β-actin. Two-way ANOVA followed by Tukey's multiple comparisons tests, genotype effect: $F(1, 80) = 27.61$, $P < 0.0001$; age effect: $F(7, 80) = 10.35$, $P < 0.0001$; interaction: $F(7, 80) = 17.32$, $P < 0.0001$. * $P < 0.05$, n = 6 mice/group.

Figure S2

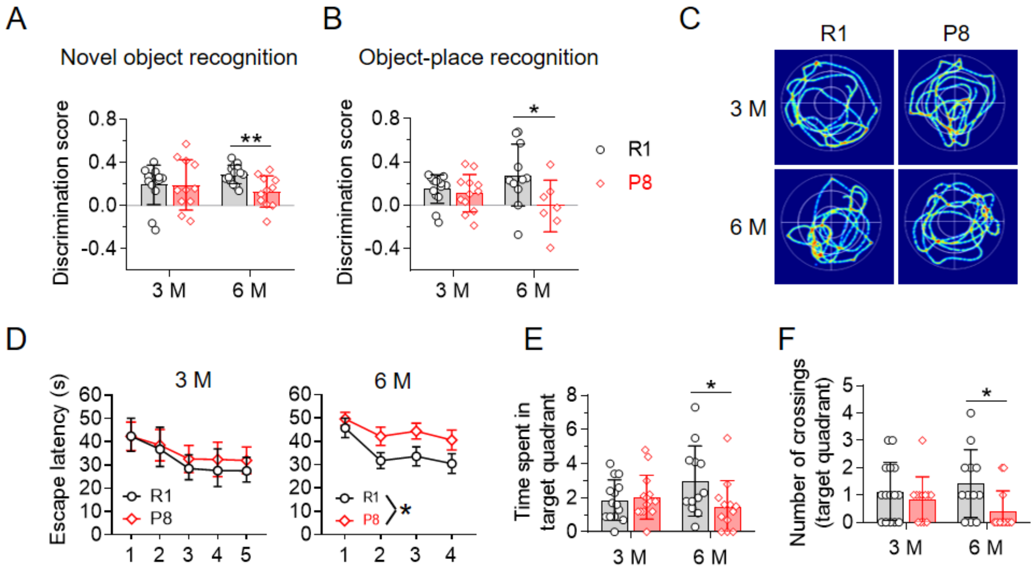

Figure S2. SAM-P8 mice show impaired spatial learning and memory at 6-month of age. (A-B) SAM-P8 mice at 6-month but not at 3-month of age showed poorer preference towards novel objects in the novel-object recognition test (A), and exhibited lower bias score towards object removed to the new locations in the object-place recognition test (B). Two-way ANOVA followed by Tukey's multiple comparisons tests, A:(genotype effect: F (1, 45) = 2.697, *P* = 0.1075; age effect F (1,45) = 0.1435, *P* = 0.7066; interaction: F (1, 45) = 2.697, *P* = 0.1075). B: (genotype effect: F (1, 40) = 6.331, *P* = 0.0160; age effect F (1,40) = 0.07076, *P* = 0.9334; interaction: F (1, 40) = 3.699, *P* = 0.0616). $^*P < 0.05$, $^{**} P < 0.01$. n = 13 or 11 mice/group. **(C)** Representative heatmaps of mice traveling during the test phase of Morris water maze tests. **(D-F)** SAM-P8 mice at 6-month but not 3-month showed impairment in both spatial learning and memory deficits in Morris water maze test, as indicated by longer escape latency during learning phase (D), and the decreased time stay (E) or number crossings in the target quadrant (F) during memory test. Repeated measures ANOVA (D) and Two-way ANOVA followed by Tukey's multiple comparisons tests (E and F). D, 3M: (genotype effect: F (1, 137) = 7.173, *P* < 0.0001; age effect F (4,137) = 0.2605, *P* = 0.9028; interaction: F (4, 137) = 0.2605, *P* = 0.9028). 6M: (genotype effect: F (1, 87) = 10.52, *P* = 0.0017; age effect F (3, 87) = 3.968, *P* = 0.0106; interaction: F (3, 87) = 0.3613, *P* = 0.7811). E: (genotype effect: F (1, 48) = 2.449, *P* = 0.1242; age effect F (1, 48) = 0.4455, *P*

= 0.5077; interaction: F (1, 48) = 3.876, *P* = 0.0548). F: (genotype effect: F (1, 48) = 5.792, *P* = 0.0200; age effect F (1, 48) = 0.08929, *P* = 0.7664; interaction: F (1, 48) = 1.749, *P* = 0.1922). * *P* < 0.05. n = 14 or 12 mice /group.

Figure S3

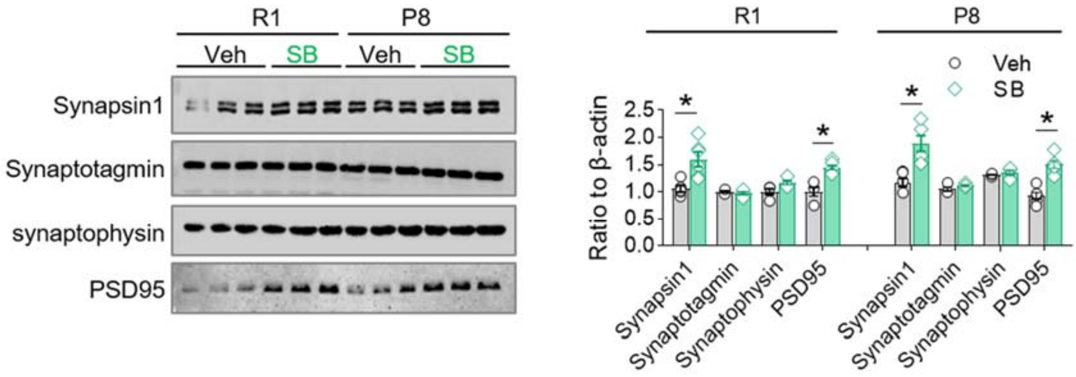

Figure S3. Inhibiting GSK-3β by SB216763 upregulates synaptic proteins in 6-month SAM-P8 and R1 mice. SB216763 upregulated the expression of synapsin1 and PSD95, but not synaptotagmin and synaptophysin, in both 6-month SAM-P8 and age-matched R1 mice. Data were normalized to β-actin. Unpaired t tests, * $P < 0.05$, n = 6 mice/group.

Tabel S1. Primer sequence of qPCR

Primer name	Primer sequence
Gsk-3β-1F	GCTGTGTGTTGGCTGAATTGT
Gsk-3β-1R	CTGCTCCTGGTGAGTCCTTT
β-catenin-1F	ACTTGCCACACGTGCAATTC
β-catenin-1R	ATGGTGCGTACAATGGCAGA
Gli1-1F	ACCAACCAACTATGGCCCTG
Gli1-1R	TAGGGTACACCCCAGCATGA
Hes1-1F	TTTTTGGCGGCTTCCAAGTG
Hes1-1R	AGGTGACACTGCGTTAGGAC
